# Geometric and dosimetric accuracy of deformable image registration between average‐intensity images for 4DCT‐based adaptive radiotherapy for non‐small cell lung cancer

**DOI:** 10.1002/acm2.13341

**Published:** 2021-07-26

**Authors:** Yulun He, Guillaume Cazoulat, Carol Wu, Christine Peterson, Molly McCulloch, Brian Anderson, Julianne Pollard‐Larkin, Peter Balter, Zhongxing Liao, Radhe Mohan, Kristy Brock

**Affiliations:** ^1^ Department of Imaging Physics The University of Texas MD Anderson Cancer Center Houston TX USA; ^2^ Department of Diagnostic Radiology The University of Texas MD Anderson Cancer Center Houston TX USA; ^3^ Department of Biostatistics The University of Texas MD Anderson Cancer Center Houston TX USA; ^4^ Department of Radiation Physics The University of Texas MD Anderson Cancer Center Houston TX USA; ^5^ Department of Radiation Oncology The University of Texas MD Anderson Cancer Center Houston TX USA

**Keywords:** 4DCT, adaptive radiotherapy, deformable image registration accuracy, non‐small cell lung cancer

## Abstract

**Purpose:**

Re‐planning for four‐dimensional computed tomography (4DCT)‐based lung adaptive radiotherapy commonly requires deformable dose mapping between the planning average‐intensity image (AVG) and the newly acquired AVG. However, such AVG‐AVG deformable image registration (DIR) lacks accuracy assessment. The current work quantified and compared geometric accuracies of AVG‐AVG DIR and corresponding phase‐phase DIRs, and subsequently investigated the clinical impact of such AVG‐AVG DIR on deformable dose mapping.

**Methods and Materials:**

Hybrid intensity‐based AVG‐AVG and phase‐phase DIRs were performed between the planning and mid‐treatment 4DCTs of 28 non‐small cell lung cancer patients. An automated landmark identification algorithm detected vessel bifurcation pairs in both lungs. Target registration error (TRE) of these landmark pairs was calculated for both DIR types. The correlation between TRE and respiratory‐induced landmark motion in the planning 4DCT was analyzed. Global and local dose metrics were used to assess the clinical implications of AVG‐AVG deformable dose mapping with both DIR types.

**Results:**

TRE of AVG‐AVG and phase‐phase DIRs averaged 3.2 ± 1.0 and 2.6 ± 0.8 mm respectively (*p* < 0.001). Using AVG‐AVG DIR, TREs for landmarks with <10 mm motion averaged 2.9 ± 2.0 mm, compared to 3.1 ± 1.9 mm for the remaining landmarks (*p* < 0.01). Comparatively, no significant difference was demonstrated for phase‐phase DIRs. Dosimetrically, no significant difference in global dose metrics was observed between doses mapped with AVG‐AVG DIR and the phase‐phase DIR, but a positive linear relationship existed (*p* = 0.04) between the TRE of AVG‐AVG DIR and local dose difference.

**Conclusions:**

When the region of interest experiences <10 mm respiratory‐induced motion, AVG‐AVG DIR may provide sufficient geometric accuracy; conversely, extra attention is warranted, and phase‐phase DIR is recommended. Dosimetrically, the differences in geometric accuracy between AVG‐AVG and phase‐phase DIRs did not impact global lung‐based metrics. However, as more localized dose metrics are needed for toxicity assessment, phase‐phase DIR may be required as its lower mean TRE improved voxel‐based dosimetry.

## 1 INTRODUCTION

Recent advances in radiotherapy (RT) have enabled the use of highly conformal treatment plans.^1^, ^2^ However, over the treatment course, patient's breathing pattern can change,^3^ and the tumor and normal tissue can change in volume, shape, and position in response to the treatment.^4^ As a result, the original treatment plan may not be optimal to deliver the prescribed dose, potentially leading to tumor underdosing and healthy tissue overdosing. To account for these changes over the treatment course, the treatment plan must be adapted to the new anatomy as previous studies have demonstrated improved clinical outcomes with lung adaptive RT.^5^, ^6^ The goal of adaptive RT is to maintain target coverage and normal tissue sparing by re‐optimizing the treatment plan, based on the most recent imaging data that reflect setup differences, patient anatomy changes, and tumor response.^7^, ^8^ An essential part of adaptive RT workflow is to consider the dose received in the initially completed fractions in the dose optimization based on the newly acquired planning image.^9^

In many RT practices for non‐small cell lung cancer (NSCLC), four‐dimensional computed tomography (4DCT) is used for treatment planning to account for respiratory‐induced motion during beam delivery.^10^ Composed of 3‐dimensional CT images acquired over full breathing cycles, a 4DCT is usually binned into 10 breathing phases, ranging from end‐inhalation phase (T0) to mid‐ventilation phase (T3) to end‐exhalation phase (T5).^11^ An average‐intensity image (AVG) can also be created by averaging pixel intensity values of all breathing phases of a 4DCT, and many lung RT practices calculate the radiation dose for treatment planning using AVG because it is composed of the average density seen at each voxel location,^12^, ^13^, ^14^ which allows the delineation of the internal target volume, comprising of individual target volumes at all phases.^15^, ^16^, ^17^ The current NRG lung template mandates that dose calculation is performed on AVG for free breathing lung cases.^18^

During plan adaptation, calculation of the composite dose requires the dose distribution on the AVG of the original planning 4DCT to be mapped onto the dose distribution on the AVG of the newly acquired 4DCT.^19^ This dose mapping can be achieved with deformable image registration (DIR) between the associated AVGs (AVG‐AVG DIR).

Intuitively speaking, accurate DIR is the prerequisite for accurate dose deformation for plan adaptation, and for 4DCT‐based NSCLC treatment planning, it is important to quantify the uncertainty in DIR between the primary and adaptive 4DCTs. Although numerous studies have evaluated the accuracy of intra‐4DCT DIR (e.g. between T0 and T5),^20^, ^21^, ^22^, ^23^, ^24^ very limited number of studies investigated the accuracy of inter‐4DCT (phase‐phase) DIR to analyze the effect of longitudinal anatomic changes over the course of RT.^25^ The RTOG 1106 trial implemented AVG‐based planning for free‐breathing treatments and rigidly registered the original and mid‐treatment AVGs for dose propagation because the uncertainty of AVG‐AVG DIR was unknown.^26^ Therefore, the current work evaluated the accuracy of AVG‐AVG DIR to provide the uncertainty within this adaptive treatment planning workflow for NSCLC.

In this study, the geometric accuracy of AVG‐AVG DIR was quantified using corresponding anatomic landmarks and was subsequently compared with the geometric accuracy of DIR between corresponding phase pairs of the 4DCTs. The accuracy of AVG‐AVG DIR and phase‐phase DIR was also evaluated with respect to landmark motion observed for each patient during standard respiratory motion at the planning stage. Finally, by evaluating the dosimetric impact of such geometric accuracy, implications of AVG‐AVG deformable dose mapping for adaptive re‐planning were investigated and the associated clinical guidelines were provided.

## 2 METHODS AND MATERIALS

### 2.1 Patient data

The original planning 4DCT and a mid‐treatment 4DCT (approximately four weeks into treatment to mimic an adaptive RT setting) were retrospectively evaluated for 28 randomly selected patients, who were previously treated with intensity‐modulated RT under an Institutional Review Board–approved randomized clinical trial for locally advanced NSCLC.^13^ Weekly 4DCTs, 6–8 per patient, were acquired during the treatment course for motion and target re‐assessment. These 4DCTs were acquired with voxel size of 0.98 × 0.98 × 2.5 mm^3^ using the Discovery CT system (General Electric Healthcare, Waukesha, WI) with 120 kVp, operated in cine mode. Patient surface motion was monitored by Varian's Real‐time Position Management Respiratory Gating (Varian Medical Systems, Palo Alto, CA).^27^

### 2.2 Hybrid intensity‐based DIR

The DIR algorithm used for this evaluation was ANACONDA (ANAtomically CONstrained Deformation Algorithm), a hybrid intensity–based DIR algorithm commercially available in RayStation v9 (RaySearch Laboratories, Stockholm, Sweden).^20^ The image registration process is as follows. Prior to image deformation, reference and target images are rigidly registered based on image similarity measured over all voxels that are enclosed by the external body contours in both images. The optimization process of ANACONDA is based on image similarity as measured by a correlation coefficient and is solved by a quasi‐Newton algorithm.^20^ This process is regularized by minimizing the Dirichlet energy of the generated deformation vector field (DVF). DVF smoothness is maintained by penalizing large shape deviations of regions of interest (ROIs) defined in the reference image, and invertibility is checked by the determinant of the Jacobian. When the optimization process is constrained by user‐delineated organ contours in both reference and target images, such geometric information makes the ANACONDA method hybrid.

In the current work, rigid registration was first established between the planning AVG and the mid‐treatment AVG, focusing on bone and tumor regions and discarding rotations to mimic the daily kilovolt alignment. With ANACONDA, T0, T3, T5, and AVG of the mid‐treatment 4DCT were registered to their counterparts of the planning 4DCT (as the reference image). Left and right lung boundaries were manually contoured in each image pair to guide the DVF as controlling ROIs. The rigid and deformable registrations were systematically performed through RayStation scripting. For each registration, the results were qualitatively assessed via image fusion of the deformed target image and the reference image with a focus on the bronchial and vascular alignment, per recommendation of the American Association of Physicists in Medicine (AAPM) Task Group 132.^28^

### 2.3 Quantitative metrics for DIR accuracy assessment

#### 2.3.1 Dice similarity coefficient

American Association of Physicists in Medicine Task Group 132 recommends metrics including Dice similarity coefficient (DSC) and target registration error (TRE) for validation of DIR accuracy. DSC is a measure of overlap between the ROI in the reference image and the same ROI in the deformed target image:

(1) $$\begin{array}{c} DSC=\frac{2*(V_{\mathrm{deformed}}\cap V_{reference})}{V_{\mathrm{deformed}}+V_{reference}} \end{array},$$

where *v_deformed_* is the volume of the deformed ROI and *v_reference_* is the volume of the reference ROI.^29^ DSCs of lung contours across all DIRs were obtained.

#### 2.3.2 Target registration error

Unlike DSC, which focuses on the alignment of the organ contour, TRE addresses the internal alignment of images. TRE is defined as the three‐dimensional Euclidian distance between a landmark's position in the deformed target image and its location in the reference image:

(2) $$\begin{array}{c} TRE=\sqrt{{\left(x_{\mathrm{deformed}}‐x_{reference}\right)}^{2}+{\left(y_{\mathrm{deformed}}‐y_{reference}\right)}^{2}+{\left(z_{\mathrm{deformed}}‐z_{reference}\right)}^{2}} \end{array},$$

where *x*, *y*, and *z* are the Cartesian coordinates in the reference image space. In the current work, vessel bifurcations were used as landmarks because of their abundance in the lungs and high contrast against air‐like lung tissue. Landmark pairs were identified on corresponding phases (T0, T3, and T5) of the planning 4DCT and the mid‐treatment 4DCT. These landmark pairs were overlaid onto the AVGs of the corresponding 4DCTs to assess the accuracy of AVG‐AVG DIR, given that the AVG comprises all phases and exists in the same image space with phases of the same 4DCT. As a result, TRE of each landmark pair was computed for AVG‐AVG DIR and phase‐phase DIRs (T0‐T0 DIR, T3‐T3 DIR, or T5‐T5 DIR).

### 2.4 Automatic landmark identification method

Manual landmark identification is a cumbersome process that could introduce uncertainties such as inter‐observer variability. For the current work, the following in‐house fully‐automatic landmark identification workflow was used.^30^ By thresholding the reference and target images, vessels on these images were automatically segmented, through which the centerlines could be extracted and the bifurcations on the centerlines detected using the neighbors’ count. The segmented vessels on the reference and target images were then registered via a separate intensity‐based Demons DIR algorithm,^31^ after which bifurcations <4 mm apart after the deformation were considered landmark pairs. Landmarks in both lungs were automatically identified on T0 pairs, T3 pairs, and T5 pairs. This workflow has been previously validated against 10 pairs of T0/T5 lung 4DCTs, each with 300 manually identified landmark pairs (DIR‐Lab, http//www.dir‐lab.com) and 10 pre‐/post‐RT pairs of liver contrast‐enhanced CTs with five manual landmarks each.

**FIGURE 1:**
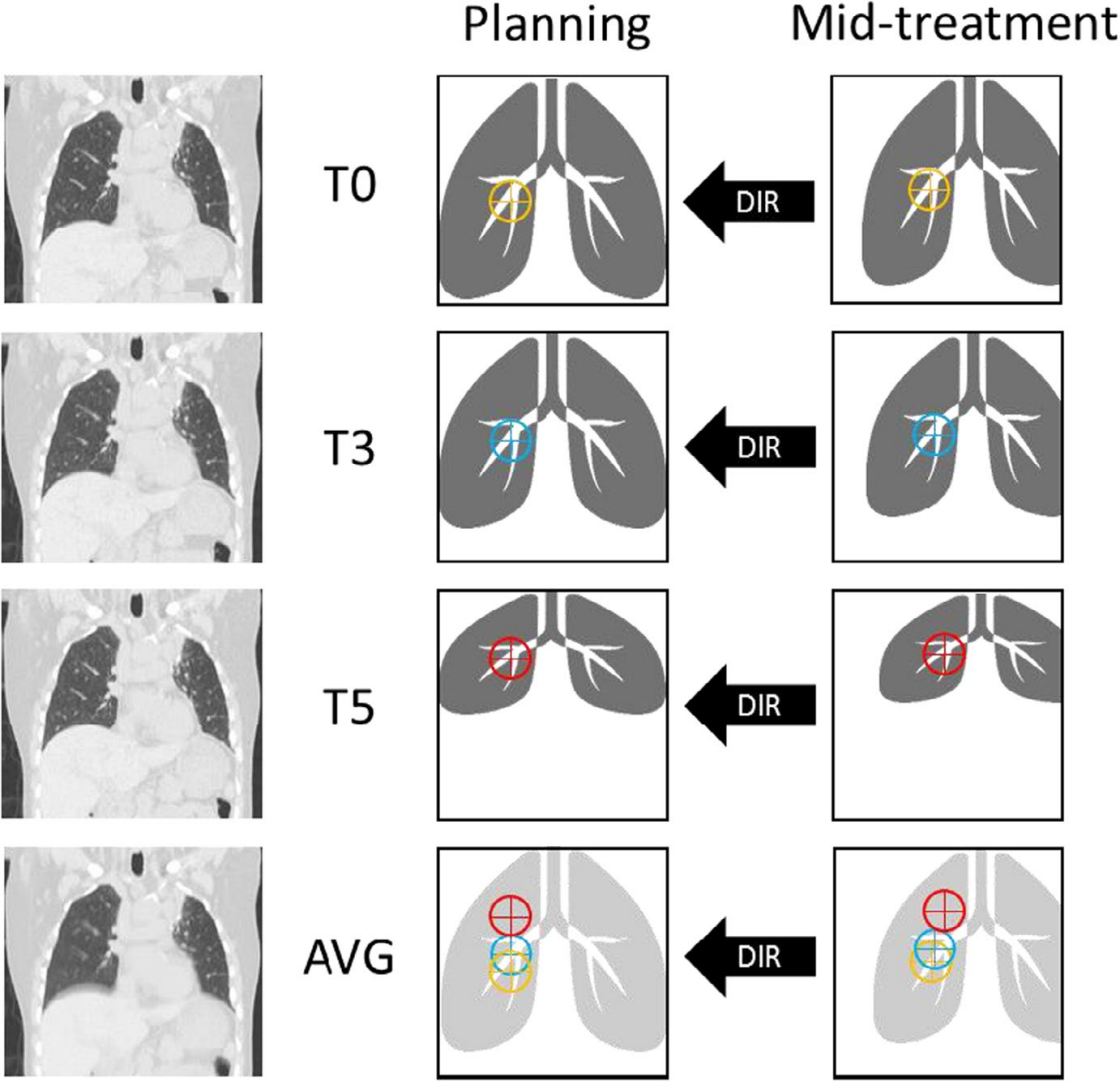
Manual landmark identification process to validate the automatic landmark workflow. The planning four‐dimensional computed tomography (4DCT) and the mid‐treatment 4DCT are shown. Coronal views of the end‐inhalation phase (T0), mid‐ventilation phase (T3), and end‐exhalation phase (T5), as well as the average‐intensity image (AVG), are shown in the left column. Simplified cartoons of the two 4DCTs are shown on the right. Deformable image registrations (DIRs) were established between corresponding phases and AVGs of the two 4DCTs. A landmark at the lower vessel bifurcation in the right lung was identified with different colors in different phases. The landmark's locations in these phases were transferred onto the AVG of each corresponding 4DCT

In the current work, the automatic workflow was also validated against manually identified landmarks in the ipsilateral lung from a random subset of 10 patients. Compared with the contralateral lung, the ipsilateral lung could inherently exhibit larger anatomic changes between the two 4DCTs owing to tumor response. For the validation, 16 anatomic landmark pairs were manually identified at vessel bifurcations in the ipsilateral lung in T0 pairs. These landmarks were uniformly distributed along the superior‐inferior direction of the lung to potentially cover a wide range of respiratory motion exhibited by different regions of the lung.^3^, ^32^ The same 16 landmark pairs were then re‐identified in the T3 pairs and T5 pairs. Each landmark (position) was then directly copied to the AVG pairs, the same way as in the automatic workflow. In total, for each 4DCT set, 16 landmarks were identified in each of the three phases, and three variations of these 16 landmark positions (48 total) were identified in the AVG. The above process is described in Figure 1. Linear regression was used to compare mean TRE values from each phase‐phase DIR of manual landmarks against the mean TRE values of automatic landmarks.

### 2.5 Landmark‐based DIR accuracy measurement

#### 2.5.1 Comparison of geometric accuracy between AVG‐AVG DIR and phase‐phase DIRs

The TRE of each landmark pair from T0‐T0 DIR, T3‐T3 DIR, T5‐T5 DIR, and AVG‐AVG DIR was obtained for all 28 patients. Paired Student's *t*‐test was used to compare TRE of AVG‐AVG DIR and TRE of T0‐T0 DIR, based on T0‐T0 landmark pairs, and such comparison was repeated for T3‐T3 landmark pairs and T5‐T5 landmark pairs. The same statistical testing was also used for comparisons among the phase‐phase DIRs.

#### 2.5.2 Effect of breathing motion

For each patient, respiratory‐induced landmark breathing motion was represented by the displacement of landmarks under hybrid intensity‐based DIR between T5 (reference exam) and T0 of the planning 4DCT, with both lungs as controlling ROI. Assuming TREs of landmark pairs in the AVG pair and corresponding phase pairs follow a Gaussian distribution, we used linear regression to quantify the effect of landmark breathing motion on TRE for AVG‐AVG DIR and T5‐T5 DIR. A more simplified comparison was made in which these landmarks were divided using a cutoff of motion magnitude of 10 mm, and a two‐sample unequal variance *t*‐test was used to compare the average TRE values for landmarks that showed at least 10 mm motion and those that showed <10 mm motion, for both AVG‐AVG DIR and T5‐T5 DIR.

#### 2.5.3 Clinical impact of DIR geometric accuracy on dose mapping

Hybrid intensity‐based DIR was established between AVGs and between T5s (from planning to mid‐treatment) with both lungs as controlling ROIs. Accordingly, the original planned dose on AVG, calculated with RayStation's default uniform dose grid of 3 mm, was deformed to the mid‐treatment 4DCT to simulate adaptive RT with both AVG‐AVG DIR and T5‐T5 DIR (warranted by AVG and phase existing in the same image space). For the ipsilateral lung, multiple linear regression was performed to correlate AVG‐AVG DIR TRE, along with several planning metrics, to the absolute difference in mean lung dose (MLD) between the planned dose deformed with AVG‐AVG DIR versus with T5‐T5 DIR, and the absolute difference in the volume of lung receiving at least 20 Gy (V_20_) between the planned dose deformed with AVG‐AVG DIR versus with T5‐T5 DIR, as clinical endpoints. The planning metrics include gross tumor volume (GTV) motion in the superior‐inferior direction, GTV volume inside lung, GTV center of gravity to diaphragm, diaphragm breathing motion in the superior‐inferior direction, GTV dose homogeneity index (DHI) (concept proposed by Ding et al.^33^), DHI of normal lung (lung excluding GTV), and percent of primary tumor volume (PTV) in normal lung. In addition, the dose discrepancy on a voxel/sub‐regional level was evaluated using the percentage of landmarks on T5 that had at least 2 Gy absolute dose difference when the dose distribution was mapped using AVG‐AVG DIR versus T5‐T5 DIR.

## 3 RESULTS

AVG‐AVG DIR and phase‐phase DIR were successfully performed for all patients. On average, 654 ± 162, 603 ± 186, and 606 ± 194 landmarks were identified on each patient's T0, T3, and T5 pairs respectively. The average DSC of left and right lung contours combined was 0.95 ± 0.02 for AVG‐AVG DIR and 0.93 ± 0.03 for all phase‐phase DIRs. AAPM Task Group 132 considers DSC of 0.80–0.90 satisfactory within the contouring uncertainty of the structures.^28^ Therefore, according to this metric, the DIRs were deemed successful.

### 3.1 Validation of automatic landmark identification workflow

**FIGURE 2:**
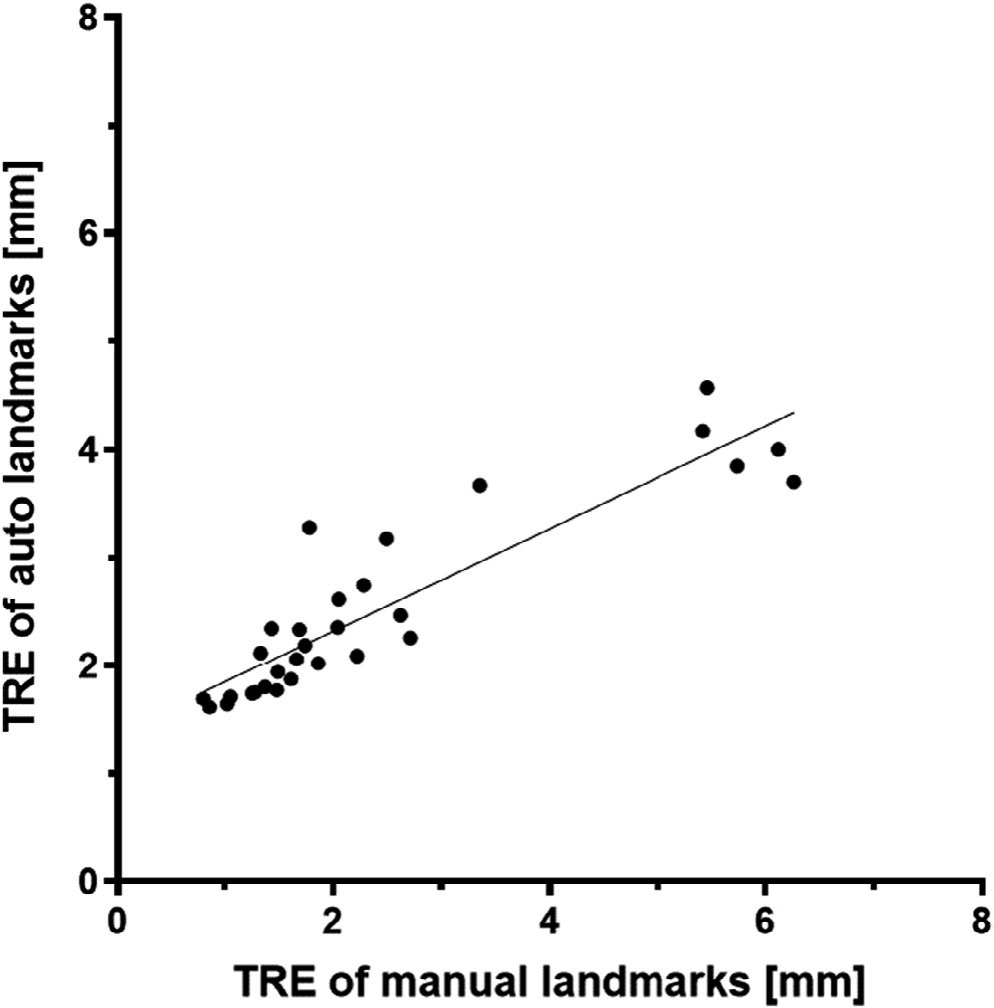
Target registration error (TRE) differences between deformable image registration (DIR) across average‐intensity images and DIR across phases for each patient. Each data point represents a labeled patient. The result of linear regression is TRE (auto)=0.5 × TRE (manual) +1.4. Coefficient of determination is 0.81

For 10 randomly selected patients, 16 landmarks were manually identified where they were uniformly distributed along the superior‐inferior direction within the ipsilateral lung. TREs of these landmarks were compared against those of the automatic landmarks for each phase‐phase DIR (Figure 2). With a slope of greater than 1.0, the automatic method consistently reported a larger TRE compared with the manual method (average TRE 2.5 ± 0.9 mm for automatic compared with 2.4 ± 1.7 mm for manual). However, the coefficient of determination was 0.8, and the averaged difference between the two methods was 0.1 mm, substantially smaller than the largest voxel dimension (2.5 mm). In addition, the paired Student's *t*‐test showed no statistical significance (*p* = 0.55), indicating that the automatic method was an acceptable substitute for the manual method.

### 3.2 AVG‐AVG DIR compared with phase‐phase DIRs

**FIGURE 3:**
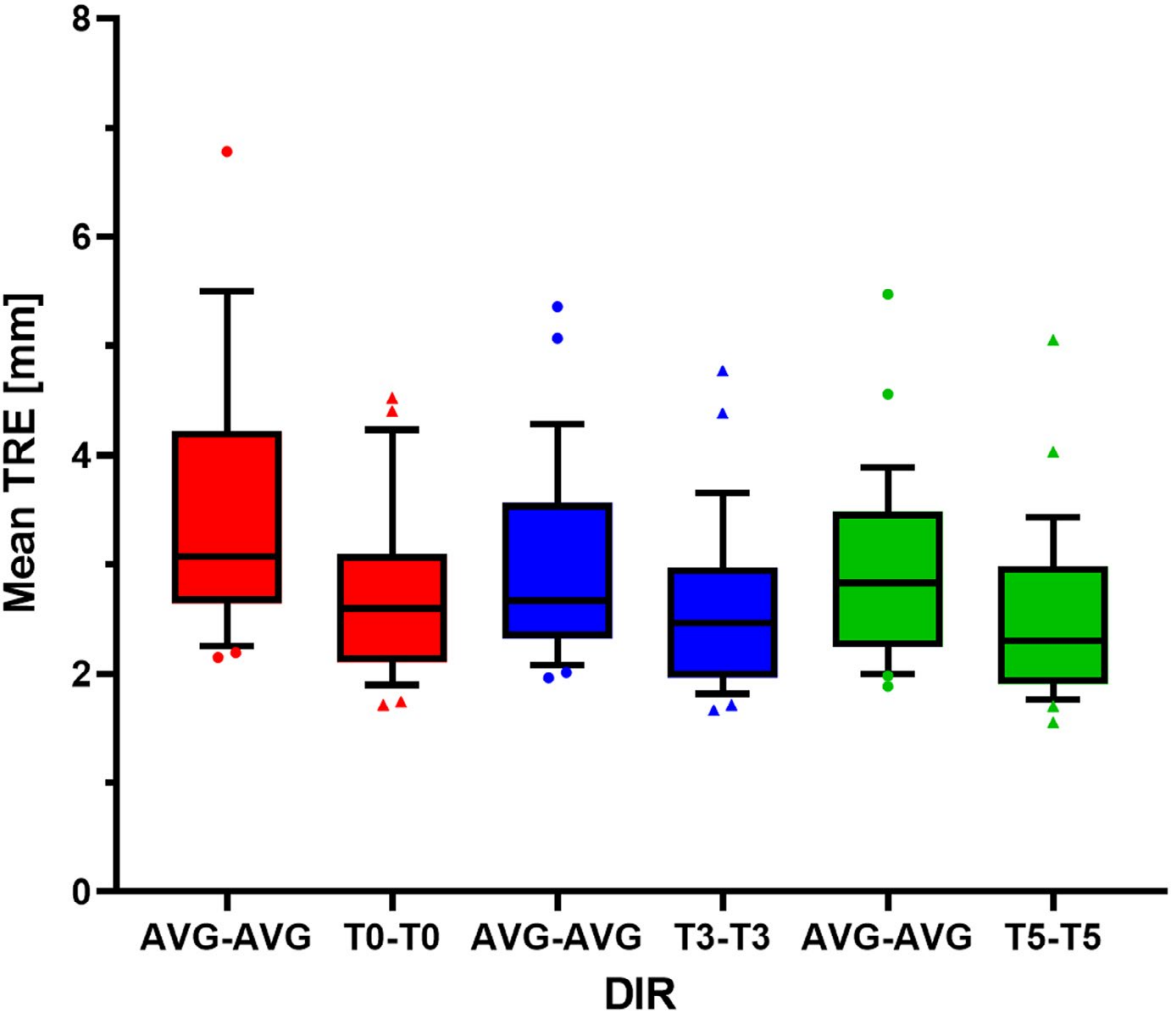
Boxplots of mean target registration error (TRE) of the 28 patients. Each color pair of boxplots represents the mean TRE of phase pairs for the corresponding phase‐phase DIR and for AVG‐AVG DIR. The standard deviation of TRE ranged from 1.0 to 3.4 mm (not shown). *AVG, average‐intensity image; DIR, deformable image registration; T0, end‐inhalation phase; T3, mid‐ventilation phase; T5, end‐exhalation phase

As shown in Figure 3, the mean TRE of T0 landmark pairs was 3.6 ± 1.1 mm for AVG‐AVG DIR compared with 2.8 ± 0.8 mm for T0‐T0 DIR (*p* < 0.001). The mean TRE of T3 landmark pairs was 3.0 ± 0.9 mm for AVG‐AVG DIR compared with 2.6 ± 0.8 mm for T3‐T3 DIR (*p* < 0.001). The mean TRE of T5 landmark pairs was 3.0 ± 0.8 mm for AVG‐AVG DIR compared with 2.5 ± 0.8 mm for T5‐T5 DIR (*p* < 0.001). In total, AVG‐AVG DIR resulted in a mean TRE of 3.2 ± 1.0 mm compared with 2.6 ± 0.8 mm for phase‐phase DIRs (*p* < 0.001). For all patients except one, TRE for AVG‐AVG DIR was higher than TRE for phase‐phase DIRs (for such patient, the TRE for AVG‐AVG DIR was 0.07 mm lower than that for T0‐T0 DIR and 0.03 mm lower than that for T3‐T3 DIR).

### 3.3 Geometric impact of landmark motion on DIR TRE

Respiratory‐induced landmark motion was represented by the displacement of T5 landmarks under T5‐T0 DIR of the planning 4DCT. Linear regressions between landmark motion and TRE for AVG‐AVG DIR as well as for T5‐T5 DIR both yielded coefficient of determination values of <0.1. When comparing landmarks grouped using a cutoff of 10 mm motion, for AVG‐AVG DIR, the mean TRE of T5 landmarks with <10 mm motion was 2.9 ± 2.0 mm compared with 3.1 ± 1.9 mm for T5 landmarks with at least 10 mm motion (*p* < 0.001), whereas for phase‐phase DIR, the mean TRE of T5 landmarks with <10 mm motion was 2.5 ± 2.0 mm compared with 2.5 ± 1.9 mm for T5 landmarks with at least 10 mm motion (*p* = 0.30). Therefore, landmark pairs with at least 10 mm motion had significantly larger TREs than those with <10 mm motion in AVG‐AVG DIR, which was not observed for phase‐phase DIR.

### 3.4 Clinical impact of DIR geometric accuracy on dose mapping

**FIGURE 4:**
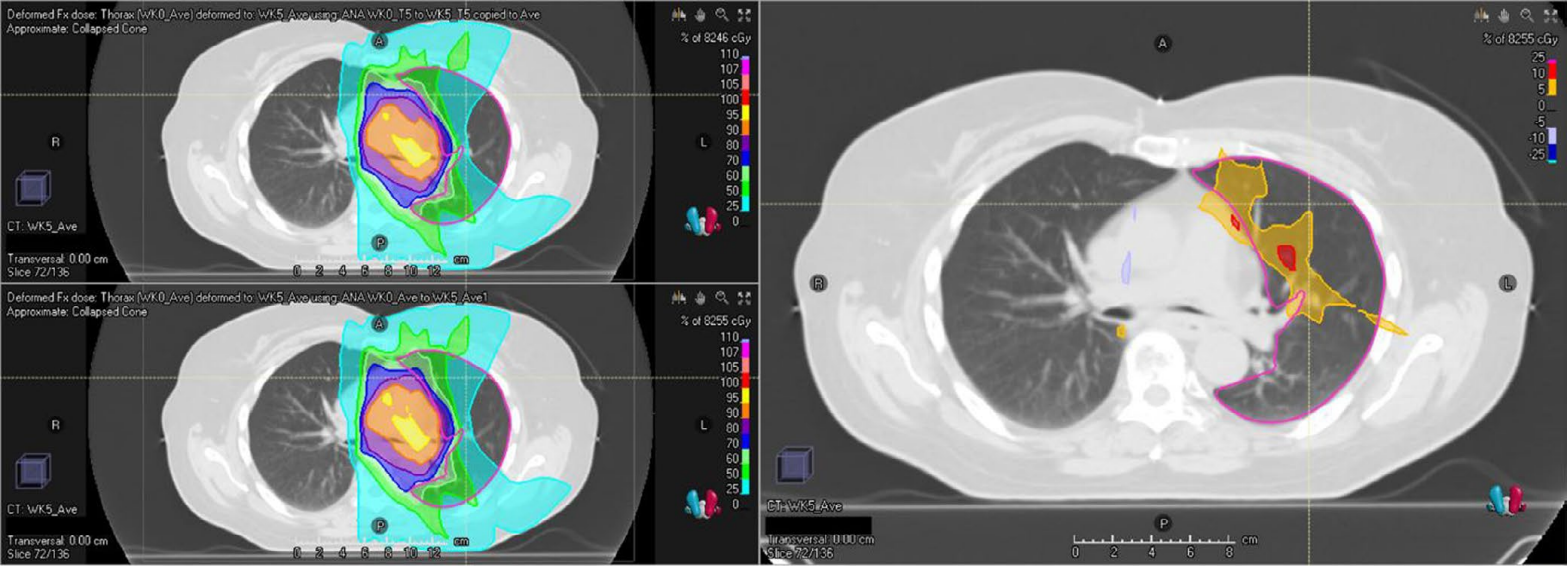
This figure shows, for patient 14, deformed doses (left panels) and their difference (right panel) on an axial slice of the average‐intensity image (AVG) of the mid‐treatment week. The upper‐left and lower‐left panels show the deformed planned dose with AVG‐AVG DIR and with T5‐T5 DIR respectively. *DIR, deformable image registration; T5, end‐exhalation phase

Figure 4 shows for patient #14 the comparison of the deformed planned dose with AVG‐AVG DIR versus T5‐T5 DIR. This patient carried the largest absolute difference in MLD between the two deformed doses.

Ipsilateral lung planning information for all 28 patients is shown in Table 1. Multi‐linear regression resulted in non‐significant correlations (*p* > 0.05) when correlating AVG‐AVG DIR TRE as well as clinical planning information (columns 3–9) to the difference in global clinical metrics of MLD and V_20_ between doses mapped with AVG‐AVG DIR versus T5‐T5 DIR (columns 11 and 12). However, when comparing these two mapped doses on a sub‐regional level, both TRE of AVG‐AVG DIR and GTV motion reached statistical significance (*p* = 0.04 and *p* = 0.01, respectively), and percent of PTV in normal lung achieved *p* = 0.06, when correlated against the metric: portion of T5 landmarks that had at least 2 Gy absolute dose difference (≥2 Gy%) (column 10). For this local dose metric, 12 patients achieved at least 10% (at least 10% of total T5 landmarks had at least 2 Gy absolute dose difference). Eleven of these 12 patients met at least one of the following conditions: TRE larger than 3.0 mm (recommended dose grid size, per AAPM Task Group 132^28^), GTV motion larger than 5.0 mm (AAPM Task Group 76^34^ recommends that respiratory management techniques be considered when tumor motion is larger than 5.0 mm), or percent of PTV in normal lung larger than 20%, achieving sensitivity of 0.92. Only 3 of the 16 patients with <10% of ≥2 Gy% demonstrated any of these three criteria, achieving a specificity of 0.81.

**TABLE 1:** Planning information for the ipsilateral lung of each analyzed patient

Patient	AVG‐AVG DIR TRE [mm]	Patient Characteristics							Dose Metrics		
		Diaph motion [mm]	GTV motion [mm]	GTV to Diaph [mm]	GTV vol [cm^3^]	PTV%	GTV DHI	DHI of normal lung	|ΔMLD| [cGy]	|ΔV_20_|	≥2 Gy%
1	2.0	16.0	4.4	80	9	13.4	3.5	0.9	7	0.1	2.1
2	2.3	7.6	1.5	79	32	11.0	4.2	0.9	7	0.0	9.6
3	1.8	5.1	1.7	73	25	8.4	2.4	0.9	20	0.3	0.0
4	2.2	19.5	4.0	124	5	3.1	2.6	0.5	7	0.4	4.8
5	2.6	10.4	6.2	20	19	13.8	2.9	0.8	55	0.8	16.5
6	2.4	10.2	0.8	56	90	18.1	1.8	1.0	15	0.1	0.4
7	2.7	5.5	0.6	85	9	10.4	2.2	0.9	37	0.6	6.3
8	5.7	4.5	0.9	76	21	5.5	3.5	0.5	5	0.1	2.1
9	1.7	14.7	0.6	35	29	12.4	2.4	1.0	20	0.3	3.2
10	3.1	27.3	3.7	27	180	8.6	4.6	0.9	80	2.0	16.6
11	2.1	8.3	1.4	19	12	12.1	2.5	0.9	11	0.1	4.3
12	3.1	5.5	1.9	32	193	24.1	4.0	1.0	16	0.1	6.0
13	1.9	26.5	0.9	81	8	7.1	2.6	0.7	1	0.0	2.1
14	4.8	5.0	0.5	12	18	7.3	2.6	0.8	84	1.6	15.5
15	2.2	6.2	4.0	90	1	8.1	2.7	0.8	28	0.9	17.8
16	4.7	9.3	0.4	93	363	13.4	3.0	1.0	18	0.2	12.2
17	2.7	10.4	1.7	80	192	23.4	4.1	0.9	12	0.1	10.2
18	3.8	14	3.6	112	235	12.6	4.2	1.0	32	0.1	11.8
19	2.4	1.4	2.8	25	53	16.8	1.7	0.8	39	0.5	5.3
20	5.0	9.6	3.0	75	28	8.1	4.5	0.8	2	0.2	11.7
21	2.3	1.0	0.6	90	21	7.7	2.5	0.8	1	0.0	0.9
22	4.3	3.6	1.8	94	35	12.3	2.2	0.9	21	0.5	10.7
23	2.4	10.6	1.0	101	9	8.6	6.3	1.0	38	0.6	8.8
24	5.4	7.8	4.7	51	23	4.7	3.3	0.5	21	0.2	21.6
25	2.3	17.7	2.2	72	41	13.9	2.9	0.9	55	0.8	7.4
26	3.3	28.3	7.7	90	33	5.3	2.4	0.6	48	1.0	8.2
27	1.9	14.2	8.6	55	19	11.2	3.6	0.9	5	0.0	10.9
28	4.6	15.1	2.9	81	53	18.5	3.3	0.9	58	0.7	10.7

**Patient Characteristics columns**: Diaph motion: the largest distance between two voxels on the diaphragm between end‐exhalation phase and end‐inhalation phase along the superior‐inferior direction. GTV motion: the distance between the center of gravity of the GTVs in end‐exhalation phase and end‐inhalation phase along the superior‐inferior direction. GTV to Diaph: the distance between the center of gravity of the GTV to the diaphragm along the superior‐inferior direction. GTV vol: volume of GTV inside normal lung (lung volume excluding GTV). PTV%: percent PTV volume in normal lung.

**Dose Metrics columns**: |ΔMLD|: absolute difference in MLD between the planned dose deformed with AVG‐AVG DIR versus with T5‐T5 DIR. |ΔV_20_|: absolute difference in V_20_ between the planned dose deformed with AVG‐AVG DIR versus with T5‐T5 DIR. ≥2 Gy%: Portion of T5 landmarks with ≥2 Gy absolute dose difference between doses mapped with AVG‐AVG DIR versus T5‐T5 DIR.

Abbreviations: AVG, average‐intensity image; Diaph, diaphragm; DHI, dose homogeneity index; DIR, deformable image registration; GTV, gross tumor volume; MLD, mean lung dose; PTV, planning target volume; TRE, target registration error; V_20_, volume of lung receiving at least 20 Gy.

## 4 DISCUSSION

In the current work, we evaluated the accuracy of AVG‐AVG DIR and compared it with that of DIR between corresponding phases (T0, T3, and T5) for 4DCT‐based treatment planning in NSCLC. Based on imaging data and landmark pairs from 28 NSCLC patients, our findings indicated that AVG‐AVG DIR has larger uncertainty, i.e., inferior accuracy, compared with phase‐phase DIR, and that breathing‐induced motion degrades the accuracy of AVG‐AVG DIR more than that of phase‐phase DIR.

AAPM Task Group 132 recommends that the target TRE should be less than the maximum image voxel size.^28^ Therefore, given that 4DCTs used in the current work were 2.5 mm thick, T5‐T5 DIR achieved the AAPM‐recommended target TRE, with mean TRE of 2.5 ± 0.8 mm, and thus can be deemed clinically acceptable. For phases typically subject to larger breathing artifacts,^35^ T3‐T3 DIR and T0‐T0 DIR almost met the target TRE, with 2.6 ± 0.8 mm and 2.8 ± 0.8 mm respectively. However, with an overall mean TRE of 3.2 ± 1.0 mm, AVG‐AVG DIR did not meet the uncertainty recommendation, with more than half of the lateral resolution (0.98 mm) over the recommended threshold. In addition, our analysis of the effect of motion showed that large landmark respiratory motion (i.e., at least 10 mm) affects AVG‐AVG DIR more than phase‐phase DIR. In summary, phase‐phase DIR was more robust against breathing motion, which was not achieved by the AVG‐AVG DIR. Therefore, per AAPM Task Group 132, additional uncertainty should be assumed, depending on the clinical protocol, if AVG‐AVG DIR is used for dose mapping. This is especially true for patients who breathe with a relatively large magnitude, which can potentially introduce a large amount of uncertainty, as well as for anatomic structures that typically have a large motion, such as tumors close to the diaphragm.

Geometrically robust points, which are necessary for TRE, require a geometrically robust image. By definition, AVG is constructed from averaging the pixel intensities of all phases over the breathing cycle. As a result, anatomy on AVGs is blurred due to breathing motion and thus carries inherent uncertainty in representing the true anatomic shapes, which makes AVG not geometrically robust. Practically speaking, AVG is commonly used as the planning image for dose calculation because it captures the entire tumor movement under respiratory motion.^12^, ^36^, ^37^, ^38^ This helps to avoid overdosing normal tissue near the tumor and under‐dosing the tumor itself, which can happen with a smooth dose intensity map around the tumor. Although AVG‐AVG DIR is the more straightforward choice for clinicians, extra care must be taken when using AVG‐AVG DIR to perform dose mapping when adaptive planning is required, given the small but potentially clinically significant uncertainty. Furthermore, considering the effect of TRE on dose metrics, a TRE reduction of 1.6 mm for phase‐phase DIR used to deform and propagate the planning dose has been shown to clinically affect decision‐making in stereotactic body RT treatment planning.^39^ These findings support the potential clinical benefit of using phase‐phase DIR rather than AVG‐AVG DIR.

The major strength of the current work was the large number of landmark pairs made available by the automatic landmark identification workflow (average of 600+ per DIR). As a result, this work contributed to closing the gap on evaluating inter‐4DCT DIR in the presence of anatomy changes through internal landmark points. In addition to using landmarks to determine TRE as the internal metric, we also used DSC of the left and right lung contours as the external metric. Because the hybrid intensity‐based DIR method was constrained by matching the lung contours on the reference and target images, DSC values would naturally be large. In addition, the DSC can quantify how well the organ boundary matches, but it does not guarantee accurate modeling of internal volume.^40^ Therefore, using DSC as the sole metric to represent DIR accuracy would be inadequate, and consequently DSC was reported to supplement TRE.

In terms of landmark identification on AVG, the same anatomic landmarks were detected on the three phase images on both 4DCTs and then were directly used for AVG‐AVG DIR. The presented method that transfers landmarks defined in the breathing phases onto the AVG of the same 4DCT enabled representation of the actual position of these landmarks (the average of a landmark's locations in breathing phases) in AVG, which is difficult to identify directly, especially in regions that experience large respiratory‐induced motion.

In our validation of automated landmarks, automatic landmarks achieved larger TREs than manual landmarks. This is potentially due to landmarks identified by automatic workflows systematically produce worse DIR, so identifying landmarks throughout the entire lung would on average produce larger TREs. This result could also be attributed to a lack of one‐one correspondence between automatic landmarks and manual landmarks and the limited number of manual landmark pairs identified. A small set of landmark points has been shown to be insufficient for calculating TRE because this misrepresents the actual spatial accuracy.^41^ In fact, only 16 landmark pairs were chosen manually per patient due to the cumbersome nature of manual landmark identification, thus potentially limiting the TRE accuracy based on these landmarks.

The difference in DIR accuracy among the three phases (T0, T3, and T5) could be attributed to differences in the magnitude of breathing artifacts. At T0, the full inhale phase, the patient changes breathing direction, so this phase is most prone to breathing artifacts due to diaphragm motion. In addition, the inhale position tends to vary from breathing cycle to breathing cycle. In contrast, in T5, the patient spends a longer proportion of the breathing phase in the exhale position, thus leading to the fewest motion artifacts; vessels especially near the diaphragm are not “washed out” and can be more accurately identified. Therefore, T5‐T5 DIR has the potential to provide the highest registration accuracy among phase‐phase DIRs. This can be applied in four‐dimensional dose calculation, with phase dose mapped and summed on T5 as the gold‐standard dose calculation method for a 4DCT.^42^ Therefore, our findings can support the investigation of the differences between T5‐based four‐dimensional planned dose and accumulated dose using T5‐based four‐dimensional weekly doses with T5‐T5 DIR. Retrospective toxicity studies on a voxel or regional level are also warranted, to achieve the most accurate representation possible for the delivered dose.

In the dosimetric study, as the landmark TRE increased, the probability of the dose to an image voxel in the planning AVG being mapped to a different anatomical location in the re‐planning AVG also increased. Such effect was pronounced in the high dose–gradient region near the GTV where adjacent image voxels shared large dose differences. It was magnified when the GTV motion was large, which further increased TRE of AVG‐AVG DIR. However, their effect was lessened on global clinical metrics such as mean lung dose, where these voxel‐level dose differences cancel each other out. For instance, patient 24 had AVG‐AVG DIR TRE of 5.4 mm (largest among all patients) and achieved 21.6% in the previously defined metric ≥2 Gy%, but only achieved 21 cGy and 0.2 in absolute MLD difference and V_20_ difference respectively. On the contrary, patient 8 carried AVG‐AVG DIR TRE of 5.7 mm but only achieved 2.1% in ≥2 Gy% due to low GTV motion (0.9 mm) and low PTV in normal lung (5.5%). These results are similar to those concluded by Hardcastle et al.,^43^ where larger registration error in high dose gradient regions caused larger dosimetric uncertainty. These observations demonstrated that the clinical impact of DIR geometric accuracy, as represented by TRE, must be interpreted in the context of the patient and dosimetric parameters. Therefore, when dose is mapped with AVG‐AVG DIR versus phase‐phase (T5‐T5) DIR, global clinical metrics are unlikely to differ significantly. However, if local doses are evaluated based on the mapped dose, phase‐based mapping should be considered.

Lastly, to provide context in the clinical adaptive workflow, the following three simplified workflows in the order of increasing complexity can be considered. (1) Three‐dimensional treatment planning using AVG‐AVG DIR: original planned dose is calculated on AVG of planning 4DCT; at adaptation, the fraction‐corrected planned dose is deformed to the AVG of the newly acquired 4DCT with AVG‐AVG DIR; the adapted dose is created based on the deformed dose on the new AVG. (2) Three‐dimensional treatment planning using phase‐phase DIR: this is an alternative to (1) where a specific phase‐phase DIR is performed and used to map the dose calculated on the original AVG onto the secondary AVG for better accuracy. (3) Full four‐dimensional treatment planning using phase‐phase DIR: dose is calculated on each phase of the planning 4DCT, deformed to a reference phase, and summed over the breathing phases; the fraction‐corrected 4D planned dose on a specified phase is deformed to the corresponding specified phase of the newly acquired 4DCT with phase‐phase DIR; the adapted dose is created based on the deformed dose.^44^

## 5 CONCLUSION

In the current work, TRE was used to quantify the geometric uncertainty of DIRs between corresponding AVGs and corresponding phases for 4DCT‐based lung adaptive RT for 28 NSCLC patients. When the region of interest has respiratory‐induced motion <10 mm, AVG‐AVG DIR may provide sufficient clinical accuracy; however, when motion is at least 10 mm, extra attention is warranted and phase‐phase DIR, especially T5‐T5 DIR, is recommended. Dosimetrically, the geometric accuracy of AVG‐AVG DIR has not been shown to significantly impact global lung‐based clinical metrics. However, phase‐phase DIR may be required for advanced toxicity correlation studies that utilize local dose metrics (e.g., dose used in voxel‐based analyses).

## CONFLICT OF INTEREST

Yulun He has nothing to disclose; Dr. Cazoulat has nothing to disclose; Dr. Wu has nothing to disclose; Dr. Peterson has nothing to disclose; Dr. McCulloch has nothing to disclose; Brian Anderson has nothing to disclose; Dr. Pollard‐Larkin has nothing to disclose; Dr. Balter reports grants from RaySearch Laboratories, grants from Varian Associates, outside the submitted work; Dr. Liao has nothing to disclose; Dr. Mohan has nothing to disclose; Dr. Brock reports grants from RaySearch Laboratories, Helen Black Image Guided Cancer Therapy Research Fund and Tumor Measurement Initiative of University of Texas MD Anderson Cancer Center, during the conduct of the study. In addition, Dr. Brock has a licensing agreement with RaySearch Laboratories with royalties paid.

## AUTHOR CONTRIBUTIONS

Yulun He has made substantial contributions to conception and design, and/or acquisition of data, and/or analysis and interpretation of data, drafted the article and revised it critically for important intellectual content, and given final approval of the version to be submitted and any revised version. Dr. Cazoulat has made substantial contributions to conception and design, and/or acquisition of data, and/or analysis and interpretation of data, has participated in drafting the article or revising it critically for important intellectual content, and given final approval of the version to be submitted and any revised version. Dr. Wu has made substantial contributions to conception and design, and/or acquisition of data, and/or analysis and interpretation of data, participated in drafting the article or revising it critically for important intellectual content, and given final approval of the version to be submitted and any revised version. Dr. Peterson has made substantial contributions to conception and design, and/or acquisition of data, and/or analysis and interpretation of data, participated in drafting the article or revising it critically for important intellectual content, and given final approval of the version to be submitted and any revised version. Dr. McCulloch has made substantial contributions to conception and design, and/or acquisition of data, and/or analysis and interpretation of data, participated in drafting the article or revising it critically for important intellectual content, and given final approval of the version to be submitted and any revised version. Brian Anderson has made substantial contributions to conception and design, and/or acquisition of data, and/or analysis and interpretation of data, participated in drafting the article or revising it critically for important intellectual content, and given final approval of the version to be submitted and any revised version. Dr. Pollard‐Larkin has made substantial contributions to conception and design, and/or acquisition of data, and/or analysis and interpretation of data, participated in drafting the article or revising it critically for important intellectual content, and given final approval of the version to be submitted and any revised version. Dr. Balter has made substantial contributions to conception and design, and/or acquisition of data, and/or analysis and interpretation of data, participated in drafting the article or revising it critically for important intellectual content, and given final approval of the version to be submitted and any revised version. Dr. Liao has made substantial contributions to conception and design, and/or acquisition of data, and/or analysis and interpretation of data, participated in drafting the article or revising it critically for important intellectual content, and given final approval of the version to be submitted and any revised version. Dr. Mohan has made substantial contributions to conception and design, and/or acquisition of data, and/or analysis and interpretation of data, participated in drafting the article or revising it critically for important intellectual content, and given final approval of the version to be submitted and any revised version. Dr. Brock has made substantial contributions to conception and design, and/or acquisition of data, and/or analysis and interpretation of data, participated in drafting the article or revising it critically for important intellectual content, and given final approval of the version to be submitted and any revised version.

## CLINICAL TRIAL STATEMENT

Data used in the paper was acquired under an Institutional Review Board–approved clinical trial.

## Data Availability

The data that support the findings of this study are available on request from the corresponding author. The data are not publicly available due to privacy or ethical restrictions.

## References

[1] Vargas C, Yan DI, Kestin LL, et al. Phase II dose escalation study of image‐guided adaptive radiotherapy for prostate cancer: use of dose‐volume constraints to achieve rectal isotoxicity. Int J Radiat Oncol Biol Phys. 2005;63(1):141‐149. 10.1016/j.ijrobp.2004.12.017 16111582

[2] Ding X‐P, Zhang J, Li B‐S, et al. Feasibility of shrinking field radiation therapy through 18F‐FDG PET/CT after 40 Gy for stage III non‐small cell lung cancers. Asian Pacific J Cancer Prev. 2012;13(1):319‐323. 10.7314/APJCP.2012.13.1.319 22502693

[3] Seppenwoolde Y, Shirato H, Kitamura K, et al. Precise and real‐time measurement of 3D tumor motion in lung due to breathing and heartbeat, measured during radiotherapy. Int J Radiat Oncol. 2002;53(4):822‐834. 10.1016/S0360-3016(02)02803-1 12095547

[4] Hill RP, Bristow RG. Tumor and normal tissue response to radiotherapy. In: Tannock IF, Hill RP, Bristow RG, Harrington L, eds. The basic science of oncology, 5th. edn. New York: McGraw‐Hill Education Medical; 2016.

[5] Tvilum M, Khalil AA, Møller DS, Hoffmann L, Knap MM. Clinical outcome of image‐guided adaptive radiotherapy in the treatment of lung cancer patients. Acta Oncol. 2015;54(9):1430‐1437. 10.3109/0284186X.2015.1062544 26206515

[6] Kataria T, Gupta D, Bisht SS, et al. Adaptive radiotherapy in lung cancer: dosimetric benefits and clinical outcome. Br J Radiol. 2014;87(1038):20130643. 10.1259/bjr.20130643 24628269 PMC4075550

[7] Yan D, Vicini F, Wong J, Martinez A. Adaptive radiation therapy. Phys Med Biol. 1997;42(1):123‐132. 10.1088/0031-9155/42/1/008 9015813

[8] Kong FM, Ten Haken RK, Schipper M, et al. A phase II trial of midtreatment PET‐CT adapted radiation therapy with concurrent chemotherapy in patients with inoperable/unresectable non‐small cell lung cancer (NSCLC). Int J Radiat Oncol. 2016;96(2):E440. 10.1016/j.ijrobp.2016.06.1735

[9] Chetty IJ, Rosu‐Bubulac M. Deformable registration for dose accumulation. Semin Radiat Oncol. 2019;29(3):198‐208. 10.1016/J.SEMRADONC.2019.02.002 31027637

[10] Hugo GD, Weiss E, Sleeman WC, et al. A longitudinal four‐dimensional computed tomography and cone beam computed tomography dataset for image‐guided radiation therapy research in lung cancer. Med Phys. 2017;44(2):762‐771. 10.1002/mp.12059 27991677 PMC5912888

[11] Vedam SS, Keall PJ, Kini VR, Mostafavi H, Shukla HP, Mohan R. Acquiring a four‐dimensional computed tomography dataset using an external respiratory signal. Phys Med Biol. 2003;48(1):45‐62. 10.1088/0031-9155/48/1/304 12564500

[12] Tian Y, Wang Z, Ge H, et al. Dosimetric comparison of treatment plans based on free breathing, maximum, and average intensity projection CTs for lung cancer SBRT. Med Phys. 2012;39(5):2754‐2760. 10.1118/1.4705353 22559646

[13] Liao Z, Lee JJ, Komaki R, et al. Bayesian adaptive randomization trial of passive scattering proton therapy and intensity‐modulated photon radiotherapy for locally advanced non‐small‐cell lung cancer. J Clin Oncol. 2018;36:1813. 10.1200/JCO 29293386 PMC6008104

[14] Antony R, Lonski P, Ungureanu E, et al. Independent review of 4DCT scans used for SABR treatment planning. J Appl Clin Med Phys. 2020;21(3):62‐67. 10.1002/acm2.12825 PMC7075381 32053280

[15] Liang X, Zheng D, Mamalui‐Hunter M, et al. ITV‐based robust optimization for VMAT planning of stereotactic body radiation therapy of lung cancer. Pract Radiat Oncol. 2019;9:38‐48. 10.1016/j.prro.2018.08.005 30138747

[16] Underberg RWM, Lagerwaard FJ, Slotman BJ, Cuijpers JP, Senan S. Use of maximum intensity projections (MIP) for target volume generation in 4DCT scans for lung cancer. Int J Radiat Oncol Biol Phys. 2005;63(1):253‐260. 10.1016/j.ijrobp.2005.05.045 16111596

[17] Mohatt DJ, Keim JM, Greene MC, Patel‐Yadav A, Gomez JA, Malhotra HK. An investigation into the range dependence of target delineation strategies for stereotactic lung radiotherapy. Radiat Oncol. 2017;12(1):166. 10.1186/s13014-017-0907-8 29100548 PMC5670725

[18] Lung Cancer Atlases . Templates, & Tools. NRG Oncology. https://www.nrgoncology.org/ciro‐lung

[19] Glide‐Hurst CK, Lee P, Yock AD, et al. Adaptive radiation therapy (ART) strategies and technical considerations: a state of the ART review from NRG oncology. Int J Radiat Oncol Biol Phys. 2021;109(4):1054‐1075. 10.1016/j.ijrobp.2020.10.021 33470210 PMC8290862

[20] Weistrand O, Svensson S. The ANACONDA algorithm for deformable image registration in radiotherapy. Med Phys. 2015;42:40‐53. 10.1118/1.4894702 25563246

[21] Samavati N, Velec M, Brock K. A hybrid biomechanical intensity based deformable image registration of lung 4DCT. Phys Med Biol. 2015;60:3359. 10.1088/0031-9155/60/8/3359 25830808 PMC4418808

[22] Coselmon MM, Balter JM, McShan DL, Kessler ML. Mutual information based CT registration of the lung at exhale and inhale breathing states using thin‐plate splines. Med Phys. 2004;31(11):2942‐2948. 10.1118/1.1803671 15587645

[23] Sarrut D, Boldea V, Miguet S, Ginestet C. Simulation of four‐dimensional CT images from deformable registration between inhale and exhale breath‐hold CT scans. Med Phys. 2006;33(3):605‐617. 10.1118/1.2161409 16878564

[24] Castillo E. Quadratic penalty method for intensity‐based deformable image registration and 4DCT lung motion recovery. Med Phys. 2019;46(5):2194‐2203. 10.1002/mp.13457 30801729 PMC6510611

[25] Cazoulat G, Owen D, Matuszak MM, Balter JM, Brock KK. Biomechanical deformable image registration of longitudinal lung CT images using vessel information. Phys Med Biol. 2016;61(13):4826‐4839. 10.1088/0031-9155/61/13/4826 27273115 PMC4975156

[26] Kong F‐M, Ten Haken RK, Schipper M, et al. Effect of midtreatment PET/CT‐adapted radiation therapy with concurrent chemotherapy in patients with locally advanced non–small‐cell lung cancer: a phase 2 clinical trial. JAMA Oncol. 2017;3(10):1358‐1365. 10.1001/jamaoncol.2017.0982 28570742 PMC5674997

[27] Sonke J‐J, Belderbos J. Adaptive radiotherapy for lung cancer. Semin Radiat Oncol. 2010;20(2):94‐106. 10.1016/J.SEMRADONC.2009.11.003 20219547

[28] Brock KK, Mutic S, McNutt TR, Li H, Kessler ML. Use of image registration and fusion algorithms and techniques in radiotherapy: report of the AAPM Radiation Therapy Committee Task Group. No. 132. Med Phys. 2017;44(7):e43‐e76. 10.1002/mp.12256 28376237

[29] Dice LR. Measures of the amount of ecologic association between species. Ecology. 1945;26(3):297‐302. 10.2307/1932409

[30] Cazoulat G, Anderson B, McCulloch M, et al. Detection of vessel bifurcations in 3d images for automatic objective assessment of deformable image registration accuracy. In 2020 Joint AAPM/COMP Meeting. Virtual. 2020.Annu Meet 2020. https://w3.aapm.org/meetings/2020AM/programInfo/programAbs.php?sid=8797&aid=52991. Accessed July 8, 2021. 10.1002/mp.15163 PMC9132059 34390007

[31] Thirion J‐P. Image matching as a diffusion process: an analogy with Maxwell’s demons. Medical Image Analysis. 1998;2(3):243‐260. 10.1016/S1361-8415(98)80022-4 9873902

[32] Mageras GS, Pevsner A, Yorke ED, et al. Measurement of lung tumor motion using respiration‐correlated CT. Int J Radiat Oncol. 2004;60(3):933‐941. 10.1016/j.ijrobp.2004.06.021 15465212

[33] Ding C, Chang CH, Haslam J, Timmerman R, Solberg T. A dosimetric comparison of stereotactic body radiation therapy techniques for lung cancer: Robotic versus conventional linac‐based systems. J Appl Clin Med Phys. 2010;11(3):212‐224. 10.1120/jacmp.v11i3.3223 PMC5720432 20717090

[34] Keall PJ, Mageras GS, Balter JM, et al. The management of respiratory motion in radiation oncology, Report of AAPM Task Group 76. Med Phys. 2006;33(10):3874‐3900. 10.1118/1.2349696 17089851

[35] Li G, Caraveo M, Wei J, et al. Rapid estimation of 4DCT motion‐artifact severity based on 1D breathing‐surrogate periodicity. Med Phys. 2014;41(11):111717. 10.1118/1.4898602 25370631 PMC4241828

[36] Liao Z, Simone II CB. Particle therapy in non‐small cell lung cancer. Transl Lung Cancer Res. 2018;7(2):141‐152. 10.21037/tlcr.2018.04.11 29876313 PMC5960664

[37] Admiraal MA, Schuring D, Hurkmans CW. Dose calculations accounting for breathing motion in stereotactic lung radiotherapy based on 4D‐CT and the internal target volume. Radiother Oncol. 2008;86(1):55‐60. 10.1016/J.RADONC.2007.11.022 18082905

[38] Wijesooriya K. Importance of 4D Simulation, Planning, and Delivery. AAPM Summer School. VT: Burlington; 2014. https://www.aapm.org/education/vl/vl.asp?id=3557

[39] Samavati N, Velec M, Brock KK. Effect of deformable registration uncertainty on lung SBRT dose accumulation. Med Phys. 2015;43(1):233‐240. 10.1118/1.4938412 PMC4691248 26745916

[40] Brock KK. Uncertainties in Deformable Image Registration. AAPM Summer School. BC: Burnaby; 2011. https://www.aapm.org/meetings/2011SS/documents/BrockDeformableImage.pdf

[41] Castillo R, Castillo E, Guerra R, Johnson VE, McPhail T, Garg AK, Guerrero T, et al. A framework for evaluation of deformable image registration spatial accuracy using large landmark point sets. Physics in Medicine & Biology. 2009;54(7):1849‐1870. 10.1088/0031-9155/54/7/001 19265208

[42] Valdes G, Lee C, Tenn S, et al. The relative accuracy of 4D dose accumulation for lung radiotherapy using rigid dose projection versus dose recalculation on every breathing phase. Med Phys. 2017;44(3):1120‐1127. 10.1002/mp.12069 28019649

[43] Hardcastle N, Bender ET, Tomé WA. The effect on dose accumulation accuracy of inverse‐consistency and transitivity error reduced deformation maps. Australas Phys Eng Sci Med. 2014;37(2):321‐326. 10.1007/s13246-014-0262-0 24652578

[44] Glide‐Hurst CK, Hugo GD, Liang J, Yan D. A simplified method of four‐dimensional dose accumulation using the mean patient density representation. Med Phys. 2008;35(12):5269‐5277. 10.1118/1.3002304 19175086 PMC2673609

